# Development of a Susceptibility Index of Apple Cultivars for Codling Moth, *Cydia pomonella* (L.) (Lepidoptera: Tortricidae) Oviposition

**DOI:** 10.3389/fpls.2015.00992

**Published:** 2015-11-13

**Authors:** Neelendra K. Joshi, Edwin G. Rajotte, Clayton T. Myers, Greg Krawczyk, Larry A. Hull

**Affiliations:** ^1^Department of Entomology, Pennsylvania State University, University ParkPA, USA; ^2^Entomology, Fruit Research and Extension Center, Pennsylvania State University, BiglervillePA, USA; ^3^United States Department of Agriculture – Agricultural Research Service, Appalachian Fruit Research Station, KearneysvilleWV, USA

**Keywords:** apple cultivars, codling moth, oviposition, susceptibility, host preference, Honeycrisp, Gala, Golden Delicious

## Abstract

Codling moth (CM), *Cydia pomonella* (L.) (Lepidoptera: Tortricidae) is a major fruit feeding pest of apples. Understanding susceptibility differences of various apple cultivars to CM oviposition is an important step in developing resistant varieties as well as monitoring and management strategies for this pest in apple orchards planted with mixed-cultivars. In this context, oviposition preferences of CM for the fruits of different apple cultivars were studied in laboratory bioassays using a series of no-choice and multiple-choice tests in 2006, 2007, and 2008. In 2006 and 2007, 10 apple cultivars, *viz*., Arlet, Fuji, Gala, Golden Delicious, Honeycrisp, Pristine, Delicious, Stayman, Sunrise, and York Imperial were evaluated, while in the 2008 tests, Golden Delicious, Honeycrisp, and York Imperial were evaluated. During the 2006 tests, preferred apple cultivars for CM oviposition were Golden Delicious and Fuji, while the least preferred were Arlet, Pristine, Sunrise, and Honeycrisp. Similarly, during the 2007 tests, Golden Delicious, Fuji and Stayman remained the preferred cultivars, while Arlet, Honeycrisp, Pristine, and Sunrise remained the least preferred cultivars. In the 2008 tests, Golden Delicious and Honeycrisp were the most and least preferred cultivars, respectively. Based on the oviposition preferences from these bioassays, a susceptibility index for each cultivar was developed. This index may be used as a standard measure in cultivar evaluations in breeding programs, and may assist fruit growers and crop consultants to select the most appropriate cultivar(s) for monitoring and detecting the initial signs of fruit injury from CM in an apple orchard planted with mixed-cultivars.

## Introduction

The codling moth (CM), *Cydia pomonella* (L.) (Lepidoptera: Tortricidae), probably originating in Europe (Pashely and Bush, 1979), is a serious pest of apples worldwide (Dean, 1989; Barnes, 1991; Witzgall et al., 2008), and causes significant economic damage to pome fruits. CM is closely associated with apple, *Malus pumila* Miller (Rosaceae), however, other species belonging to various plant families, such as pears (*Pyrus* sp.), quinces (*Cydonia* *oblonga* Mill.), peaches (*Prunus persica*) (L.), wild haws (*Crataegus* sp.), English walnuts (*Juglans regia* L.) (Shelford, 1927), plum (*Prunus* sp.), nectarines (*Prunus* sp.), and sweet cherry (*Prunus avium* L.) are also reported as host plants (Madsen and Borden, 1954; Barnes, 1991).

CM completes its life cycle in four different stages, viz., egg, larva, pupa, and adult. The eggs of CM are disk-shaped, flattened, or ovate, and measure about 0.98 by 1.25 mm in diameter (Putman, 1963; Dean, 1989). Development time for eggs largely depends on temperature, and upon hatching on or near fruits, the CM larva penetrates the epidermis of the fruit, feeds on the fruit pulp and eventually making its way to the core, where the larva feeds on the seeds. After feeding on the seeds, mature larvae (fifth instar) make their way to the periphery of the fruit and make a hole to exit from the fruit. Larvae then search for a suitable place for spinning a cocoon to pupate or enter into diapause in order to avoid unfavorable environmental conditions (for instance, winter). Upon emergence, adult moths feed on the exudates from fruits and other parts of their host plants (Geier, 1963), and copulate during the dusk period (Van Leeuwen, 1929). Multiple mating occurs in both sexes (Gehring and Madsen, 1963; Howell et al., 1978), and fecundity of the female varies from host to host (Phillips and Barnes, 1975).

The fruits and leaves of apple tree release different volatiles that attract female moths to the host tree and regulate host-finding mechanisms (Wearing et al., 1973; Sutherland et al., 1974; Hern and Dorn, 1999). The main source of attraction of CM to apple trees and other host plants are kairomones (i.e., *E, E* α-farnesene and *Z, E* α-farnesene), which are naturally occurring sesquiterpene compounds (Wearing and Hutchins, 1973). Kairomones likely induce female moths to lay their eggs directly on fruit or in close vicinity of fruits and fruit clusters (Wildbolz, 1958). Oviposition in CM is mainly stimulated by a sesquiterpene compound known as α-farnesene (Wearing and Hutchins, 1973). Most eggs (up to 90%) are laid within 10 cm of a fruit (Blomfield et al., 1997). The size of fruit clusters also has significant impacts on the distribution of eggs. The number of eggs laid on fruit and nearby leaves increases with an increase in the size of the fruit cluster (Jackson, 1979; Blomfield et al., 1997). In a field environment with different apple cultivars, CM females deposit eggs on fruits, as well as both sides of spur and shoot leaves (Joshi et al., 2009; Joshi, 2011).

Female CM may discriminate among apple cultivars for oviposition as they do for other hosts such as walnut (Shelton and Anderson, 1990). The fruit size of walnut and its chemical composition varies across different commercial cultivars (Tulecke and McGranahan, 1994) and are known to affect oviposition preferences (Bezemer and Mills, 2001). In addition, the maturity level of fruits of different walnut cultivars is also known to affect the oviposition preferences of CM, as the female moths prefer to oviposit on mature rather than immature fruits (Olson, 1977; Shelton and Anderson, 1990). However, in the case of apple, such studies related to oviposition/host preference are restricted to several cultivars with very few published reports (Phillips and Barnes, 1975; Blomfield et al., 1997). Considering the importance of oviposition preferences in understanding host plant resistance, in this study we investigated susceptibility of 10 apple cultivars for CM oviposition in the laboratory. In particular, we determined if oviposition and oviposition-site preference of CM varies among apple cultivars, and if there are any differences in the susceptibility of apple cultivars for CM oviposition during the early and late crop season. Based on the results from these studies, a susceptibility index of apple cultivars for CM oviposition was developed. This index may be used as a standard measure in cultivar evaluations and breeding programs to develop future resistant varieties as well as assisting fruit growers and pest management consultants select the most appropriate cultivar(s) for monitoring and detecting the initial signs of a CM infestation.

## Materials and Methods

Over three years, a series of laboratory experiments were conducted to study the susceptibility of 10 commercial apple cultivars, *viz*., ‘Arlet,’ ‘Gala,’ ‘Golden Delicious,’ ‘Fuji,’ ‘Honeycrisp,’ ‘Pristine,’ ‘Delicious,’ ‘Stayman,’ ‘Sunrise,’ and ‘York Imperial’ for CM oviposition. Two sets of experiments, based on fruit maturity, were conducted each year with the fruits collected from trees during the second week of July and either the first or second week of August in 2006, 2007, and 2008. General descriptions of bloom time, harvest time and an estimated range of fruit maturity in days after full bloom of the apple cultivars used in this study are given in **Table 1.**

**Table 1:** Description of bloom time, harvest time and an estimated range of fruit maturity in terms of days after full bloom of apple cultivars used in multiple-choice and no-choice experiments.

Apple cultivars	Bloom time	Harvest time	DAFB^∗^ (estimate range)
Arlet	Early – midseason	Mid September	125–130
Fuji	Mid – late season	Late October – Mid November	170–185
Gala	Midseason	Late August	110–120
Golden Delicious	Midseason	Mid September – Early October	135–150
Honeycrisp	Early season	Mid September	125–140
Pristine	Early season	Early August	90–100
Delicious	Mid season	Late September	135–155
Stayman	Early season	Late October	165–175
Sunrise	Midseason	Mid August	95–105
York Imperial	Midseason	Late October	170–180


*^∗^DAFB, Days after full bloom.*

*Source: Pennsylvania Tree Fruit Production Guide (2006–2007).*

### Experimental Fruits

Fruits of all cultivars were collected from unsprayed (without insecticide application) trees (10–33 years old) in apple orchards established in south facing slopes with typical well-drained soils of the Appalachian region. Fruits were stored in small cardboard boxes in a cold room (0°C). Fruits were removed from the cold room approximately 4–5 h before the start of each experiment. All fruits were washed three times with clean cold water and were carefully inspected via a 10X Opti-Visor^®^ lens (Donegan Optical Co., Lenexa, KS, USA) for field oviposition/infestation by CM and other insects. Fruits of approximately similar size were vertically suspended in oviposition chambers by tying the stem to the top of the oviposition chamber using aluminum wire. Fruits damaged while being placed in oviposition chambers were discarded and replaced by new fruits from the same lot.

### Experimental Insects

Codling moth adults used in this study were obtained from a laboratory colony established from adults or larvae collected from a block of apples located at The Pennsylvania State University, Fruit Research and Extension Center (FREC), Biglerville, PA, USA. Green thinning apples of various cultivars were used to maintain the laboratory colony/insect culture throughout the year during this study year. Pupae were collected from rearing containers in cardboard strips, and kept in environmentally controlled chambers (18–20°C) till their use. CM pupae of similar age were selected and sexed, and placed into the oviposition chambers. The adult moths were allowed to emerge, mate, and freely oviposit on fruits. Pupae were regularly monitored for adult emergence. If there was no adult emergence from a pupa within 3 days of release, then it was replaced by an adult (2–3 days old) of the same sex from the same pupal lot.

### Experimental Design (Multiple-choice and No-choice Tests)

Multiple-choice oviposition preference tests and no-choice preference tests were conducted for both fruit maturity sets. In the no-choice tests, the oviposition chamber consisted of transparent plastic cups (1.0 L) internally lined with charcoal-colored fiberglass screen. In the multiple-choice tests, a cylindrical chamber (length = 0.81 m, diameter = 0.17 m) made of transparent fiberglass internally lined with fine aluminum mesh screening served as the oviposition chamber. In the multiple-choice tests, fruits of each cultivar were allocated to one of several locations at random in the oviposition chamber. During the study period, insects were maintained under laboratory conditions (temperature ∼21–23°C, relative humidity ∼70%, and photoperiod 11:10 h light:dark with an ∼3 h period of dim light for oviposition induction). The year-wise description of these bioassays is as follows:

#### 2006 Bioassays

Nine cultivars (‘Arlet,’ ‘Golden Delicious,’ ‘Fuji,’ ‘Honeycrisp,’ ‘Pristine,’ ‘Delicious,’ ‘Stayman,’ ‘Sunrise,’ and ‘York Imperial’) were evaluated in multiple-choice and no-choice tests during the first set (July) of experiments. In the second set of experiments (August), all cultivars (except ‘Pristine’ which was replaced by ‘Gala’) were again evaluated. Each treatment (cultivar) was replicated at least eight times in the multiple-choice tests and 10 times in the no-choice tests. All fruits were collected during 14–17 July and 12–15 August for the first set (19 July) and second set (25 August) of experiments, respectively. Fruits of all cultivars (except ‘Arlet,’ ‘Pristine,’ and ‘Sunrise’) were collected from non-insecticide sprayed trees at FREC, Biglerville. Fruits of ‘Arlet,’ ‘Pristine,’ and ‘Sunrise’ cultivars (collected from an orchard partially sprayed with common orchard pesticides for the purpose of general maintenance) were received from the USDA Appalachian Fruit Research Station, Kearneysville, WV, USA. In both no-choice experiments (early and late), one pair of unmated male and female adults was placed per cup, and the number of deposited eggs was counted after 8 days. In multiple-choice tests, seven and six pairs of unmated adults were utilized in the early and late experiments, respectively. Total numbers of eggs were counted after 15 days (early), and 10 days (late). The position of each egg on fruit (stem, calyx, or lateral) was recorded.

#### 2007 Bioassays

All 10 cultivars were evaluated in multiple-choice and no-choice tests conducted during the months of July and August. Each treatment (cultivar) had 8 and 10 replicates in the no-choice and multiple-choice tests, respectively. Fruits were collected during 12–15 July (early) 11–14 August (late). Fruits of all cultivars (except ‘Arlet,’ ‘Pristine,’ and ‘Sunrise’) were collected from non-insecticide sprayed trees at FREC, Biglerville. Fruits of ‘Arlet,’ ‘Pristine,’ and ‘Sunrise’ cultivars (collected from partially sprayed orchards) were received from the USDA Appalachian Fruit Research Station, Kearneysville, WV, USA for the early set of experiments, and for the late set of experiments from The Russell E. Larson Agricultural Research farm, Rock Springs, PA, USA. The early set of multiple-choice and no-choice tests were conducted on 17 July, while the late set of experiments were conducted on 16 August. In the no-choice tests, two pairs of unmated male and female adults were used in both sets of no-choice tests. In multiple-choice tests, three pairs of unmated male and female adults were used in both sets of multiple-choice tests. In all tests, the total numbers of deposited eggs on fruits were counted after 10 days. The position of eggs on the fruits was recorded as per the procedure used in the 2006 bioassays.

#### 2008 Bioassays

Based on the results of bioassays conducted during the first two years, only three cultivars (‘Golden Delicious,’ ‘Honeycrisp,’ and ‘York Imperial’) were further evaluated in the third year. Fruits of similar size were collected from non-insecticide sprayed trees at FREC, Biglerville, and utilized the same day for both the no-choice and multiple-choice experiments. The study was replicated 15 and 8 times in the no-choice and multiple-choice tests, respectively. In the multiple-choice tests, two fruits of each treatment/cultivar were used in each replication. In the early experiment, fruits were collected on 22 July, and used in both types of tests on the same day, and observations on eggs were taken after 10 days. In the late set, fruits were collected on 28 August, and observations were recorded after 11 days in both no-choice and multiple-choice tests. Similar to previous years, the position of eggs on the fruits was recorded in 2008.

### Statistical Analysis and Development of Oviposition-based Susceptibility Index

A general linear mixed-model analysis of variance (ANOVA) was used to analyze the data. In the analysis, two similar statistical models were used to address the study objectives. The first model (**Table 2**) was used to determine: (a) the oviposition preference of CM among apple cultivars; (b) differences in oviposition preferences during the early and late season (i.e., based on time of fruit collection: early [July] versus late season [August]); and (c) differences in oviposition preferences in the multiple-choice and no-choice tests. The second model, which includes the egg counts by position on the fruit, was used to determine CM oviposition-site preferences across different cultivars (**Table 3**). The mixed-model ANOVA analysis was performed using R software (ISBN 3-900051-07-0; R Development Core Team, 2005).

**Table 2:** Mix model ANOVA results of the sum of number of eggs per pair of codling moth and covariates (year, apple cultivar, and season [early and late]).

Covariates	*df*	*F*-value	*P-*value
Year	2	155.7609	0.000
Season	1	63.7488	0.000
Cultivar	9	49.2121	0.000
Year:Season	2	15.4542	0.000
Year:Cultivar	11	5.5816	0.000
Season:Cultivar	9	4.0555	0.000
Year:Season:Cultivar	9	2.9684	0.002
Residuals	778		


*Response variable in this analysis is eggs per pair of codling moth per fruit.*

*All data and all years pooled together.*

**Table 3:** Mix model ANOVA results of the sum of mean number of eggs per pair of codling moth and covariates (year, apple cultivar, position of eggs on apple [calyx, stem, and lateral sites], and season [early and late]).

Covariates	*df*	*F*-value	*P-*value
Site	2	523.0084	0.000
Cultivar	9	126.9134	0.000
Year	2	217.1037	0.000
Season	1	95.9429	0.000
Site:Cultivar	18	2.8307	0.000
Site:Year	4	4.1102	0.003
Cultivar:Year	11	10.6993	0.000
Site:Season	2	0.7818	0.458
Cultivar:Season	9	8.6888	0.000
Year:Season	2	17.0352	0.000
Site:Cultivar:Year	22	1.9029	0.007
Site:Cultivar:Season	18	1.4118	0.115
Site:Year:Season	4	8.6915	0.000
Cultivar:Year:Season	9	4.7172	0.000
Site:Cultivar:Year:Season	18	0.8713	0.615
Residuals	2334		


*Response variable in this analysis is eggs per pair of codling moth per fruit.*

*All data and all years pooled together.*

Oviposition preference based on the mean number of eggs (per pair of CM per fruit) was determined for each cultivar. The data sets were transformed (to achieve the assumptions of parametric analysis) by taking the natural log of the “eggs per pair” variable. Pairwise comparisons were done among all cultivars, and means were separated using Tukey’s honest significant differences *post hoc* test (*P* < 0.05) when ANOVA was significant (Zar, 1999).

The CM oviposition susceptibility index (based on oviposition preferences of CM) for each cultivar was characterized as:

$$SI=\frac{1}{n}\underset{i,j,k,t}{\Sigma }SEPP\left(i,j,k,t\right)$$

Where, *SI* = Susceptibility index; *SEPP* = Standardized mean eggs per pair of moths for an apple cultivar [*i*]; *j* = Time of fruit collection (early or late); *k* = year of observation; and *t* = type of tests (i.e., no-choice and multiple-choice tests).

Standardized mean eggs per pair of moths for an apple cultivar were determined as following:

$$SEPP[i]=\frac{EPP[i]}{{EPP}_{\max}[i]}$$

Where, *SEPP* = Standardized mean eggs per pair of moths for an apple cultivar [*i*]; *EPP* [*i*] = Mean number of eggs per pair of moths on an apple cultivar [*i*]; and *EPP*_max_ [*i*] = Maximum number of eggs per pair of moths on an apple cultivar [*i*].

Mean total number of eggs per pair of moths (EPP) on a cultivar was calculated by the following equation:

$$EPP[i]={EPP}_{C}[i]+{EPP}_{S}[i]+{EPP}_{L}[i]\left(3\right)$$

Where *EPP* [*i*] = Mean number of eggs per pair of moths on an apple cultivar [*i*]; *EPP*_C_[*i*] = Mean number of eggs per pair of moths on calyx side of an apple cultivar [*i*]; *EPP*_S_[*i*] = Mean number of eggs per pair of moths on stem side of an apple cultivar [*i*]; and *EPP*_L_[*i*] = Mean number of eggs per pair of moths on lateral side of an apple cultivar [*i*].

Oviposition susceptibility index of all cultivars was compared and means were separated using Fisher’s protected least significant differences *post hoc* test (*P* < 0.05) when ANOVA was significant (Zar, 1999). The analysis was performed using SPSS-13 statistical software (SPSS Inc., Chicago, IL, USA).

## Results

### 2006 Early Season (July)

**FIGURE 1:**
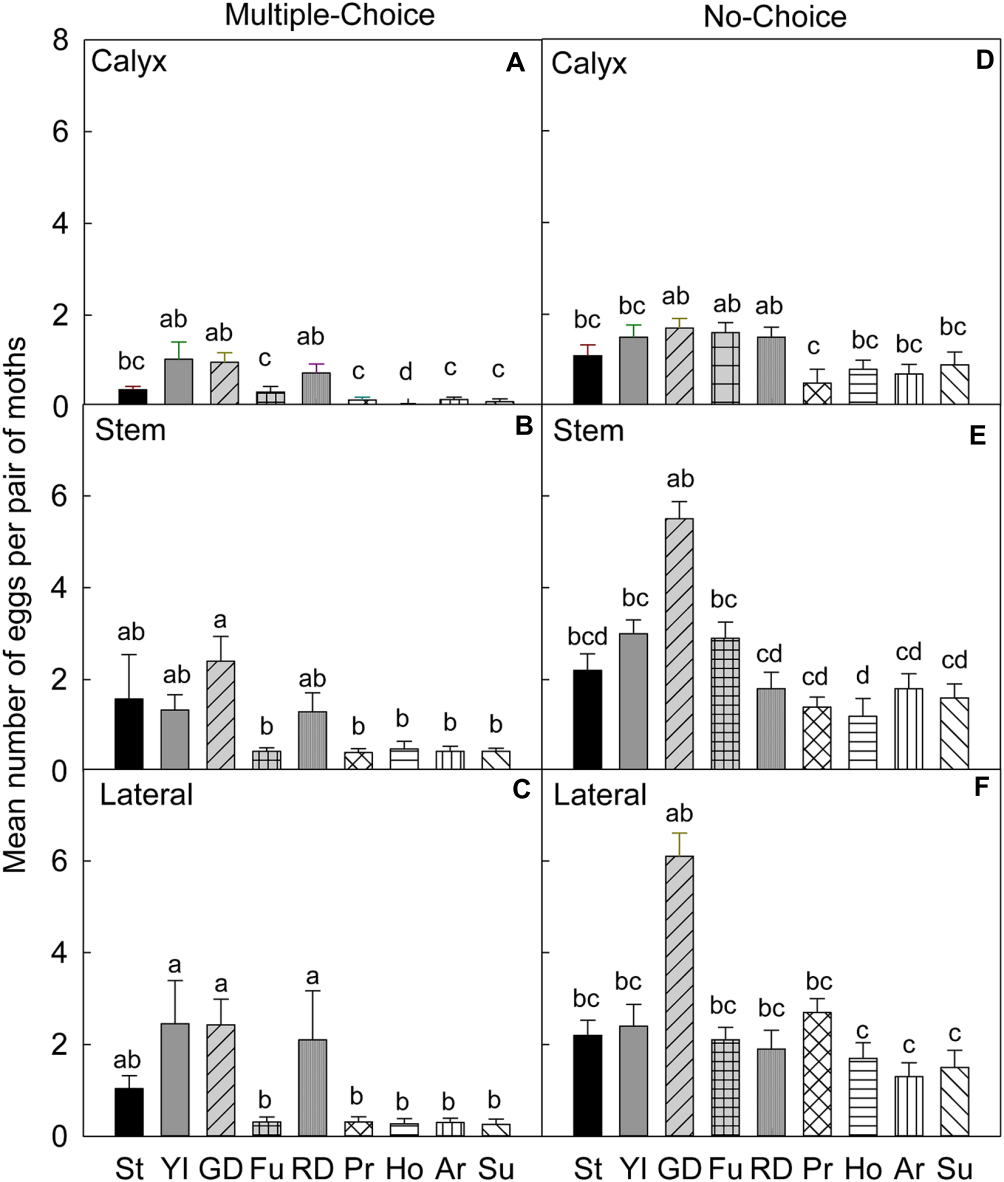
**Relative susceptibility of different apple cultivars for oviposition of codling moth during July 2006 (early season/Set 1).** Mean number of eggs per pair of moths per fruit on calyx, stem, and lateral sides of fruits of different cultivars are shown in multiple-choice tests **(A–C)** and no-choice tests **(D–F)**. St, Stayman; YI, York Imperial; GD, Golden Delicious; Fu, Fuji; RD, Delicious; Pr, Pristine; Ho, Honeycrisp; Ar, Arlet; Su, Sunrise. *N* = 8 for all the multiple-choice tests, and *N* = 10 for all the no-choice tests. Each bar represents standard error of mean. Different letters over bars indicate significant difference (*P* < 0.05).

In the multiple-choice test, on the calyx site of fruits (**Table 4**), CM females laid significantly higher numbers of eggs on ‘York Imperial,’ ‘Golden Delicious,’ and ‘Delicious’ than other cultivars (*P* < 0.05; **Figure 1A**). In contrast, the lowest numbers of eggs were laid on the ‘Honeycrisp’ cultivar (*P* < 0.05; **Figure 1A**). On the stem site of fruits, CM females laid significantly more eggs on ‘Stayman,’ ‘York Imperial,’ ‘Golden Delicious,’ and ‘Delicious’ than all other cultivars (*P* < 0.05; **Figure 1B**). On the lateral site of fruits, CM females preferred ‘York Imperial,’ ‘Golden Delicious,’ and ‘Delicious’ than the cultivars ‘Pristine,’ ‘Honeycrisp,’ ‘Arlet,’ and ‘Sunrise’ (*P* < 0.05; **Figure 1C**).

**Table 4:** Statistical details of oviposition site preferences of codling moth across different cultivars.

Year	Season/Time	Test type	*df ^∗^*					Oviposition Sites on fruits
				
				Calyx		Stem		Lateral	
						
				*F-*value	*P-*value	*F-*value	*P-value*	*F-*value	*P-*value
2006	Early	Multiple-choice	8	9.18	<0.001	5.92	<0.001	8.56	<0.001
2006	Early	No-choice	8	3.51	0.002	4.86	<0.001	2.88	0.007
2006	Late	Multiple-choice	8	3.67	0.001	8.26	<0.001	9.18	<0.001
2006	Late	No-choice	8	2.46	0.019	6.03	<0.001	6.57	<0.001
2007	Early	Multiple-choice	9	3.43	0.002	15.64	<0.001	17.12	<0.001
2007	Early	No-choice	9	5.38	<0.001	11.04	<0.001	16.57	<0.001
2007	Late	Multiple-choice	9	2.94	0.005	10.55	<0.001	12.14	<0.001
2007	Late	No-choice	9	2.16	0.032	12.37	<0.001	10.76	<0.001
2008	Early	Multiple-choice	2	8.26	0.002	15.97	<0.001	9.23	0.001
2008	Early	No-choice	2	0.76	0.474	36.87	<0.001	16.43	<0.001
2008	Late	Multiple-choice	2	7.12	0.004	14.44	<0.001	19.66	<0.001
2008	Late	No-choice	2	16.91	<0.001	29.67	<0.001	34.68	<0.001


*^∗^df* values are same across different oviposition sites.

In the no-choice test, on the calyx site (**Table 4**), the female moths significantly preferred to oviposit on ‘Golden Delicious’ (*P* = 0.009), ‘Fuji’ (*P* = 0.005), and ‘Delicious’ (*P* = 0.008), compared to ‘Pristine’ (**Figure 1D**). On the stem site, ‘Golden Delicious’ was significantly more preferred than ‘Delicious’ (*P* = 0.014), ‘Pristine’ (*P* = 0.013), ‘Honeycrisp’ (*P* < 0.001), ‘Arlet’ (*P* = 0.047), and ‘Sunrise’ (*P* = 0.023; **Figure 1E**). On the lateral site, ‘Golden Delicious’ was significantly more preferred than ‘Honeycrisp’ (*P* = 0.049), ‘Arlet’ (*P* = 0.004), and ‘Sunrise’ (*P* = 0.008; **Figure 1F**); however, the total number of eggs on ‘Golden Delicious’ was not significantly different from that of all other cultivars (*P* > 0.05; **Figure 1F**).

### 2006 Late Season (August)

**FIGURE 2:**
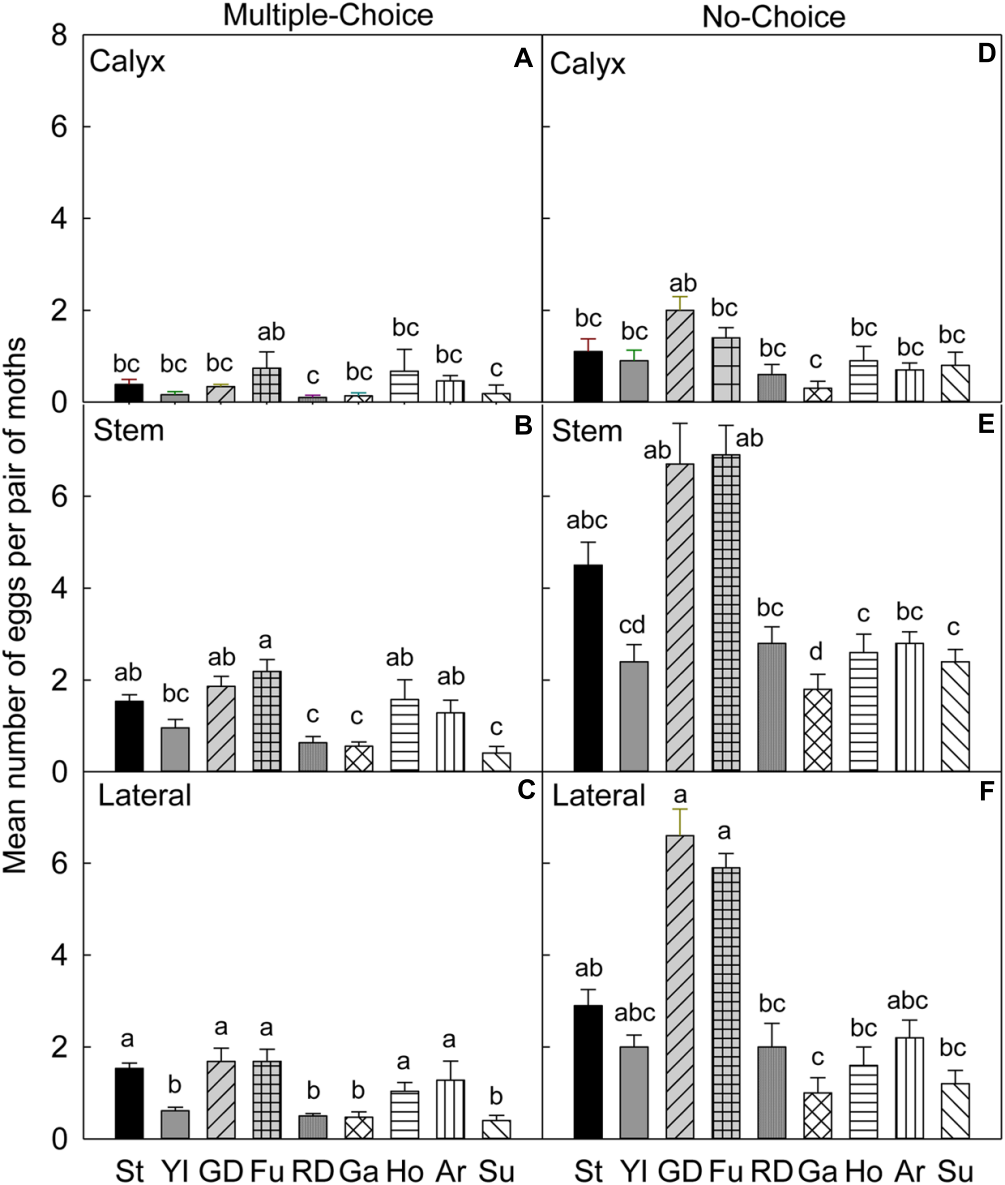
**Relative susceptibility of different apple cultivars for oviposition of codling moth during August 2006 (late season/Set 2).** Mean number of eggs per pair of moths per fruit on calyx, stem, and lateral sides of fruits of different cultivars are shown in multiple-choice tests **(A–C)** and no-choice tests **(D–F)**.St, Stayman; YI, York Imperial; GD, Golden Delicious; Fu, Fuji; RD, Delicious; Ho, Honeycrisp; Ar, Arlet; Su, Sunrise; Ga, Gala. *N* = 8 for all the multiple-choice tests, and *N* = 10 for all the no-choice tests. Each bar represents standard error of mean. Different letters over bars indicate significant difference (*P* < 0.05).

In the multiple-choice test, on the calyx site (**Table 4**), the oviposition preference of CM was not significantly different for all cultivars (*P* > 0.05), except for ‘Fuji’, when compared to ‘Delicious’ (*P* = 0.042), and ‘Sunrise’ (*P* = 0.015; **Figure 2A**). On the stem (**Figure 2B**) and lateral (**Figure 2C**) sites of fruits, ‘Stayman,’ ‘Golden Delicious,’ ‘Fuji,’ ‘Honeycrisp,’ and ‘Arlet’ rather than ‘Delicious,’ ‘Gala,’ and ‘Sunrise’ were the significantly preferred cultivars (*P* < 0.05).

In the no-choice test, on the calyx site (**Table 4**), CM deposited more eggs on ‘Golden Delicious’ than on ‘Gala’ (*P* = 0.011), otherwise, there was no significant differences between ‘Golden Delicious’ and all other cultivars (*P* > 0.05; **Figure 2D**). On the stem (**Figure 2E**) and lateral (**Figure 2F**) sites of fruits, CM deposited more eggs on ‘Golden Delicious’ and ‘Fuji’ than on ‘Gala,’ ‘Honeycrisp,’ and ‘Sunrise’ (*P* < 0.05).

### 2007 Early Season (July)

**FIGURE 3:**
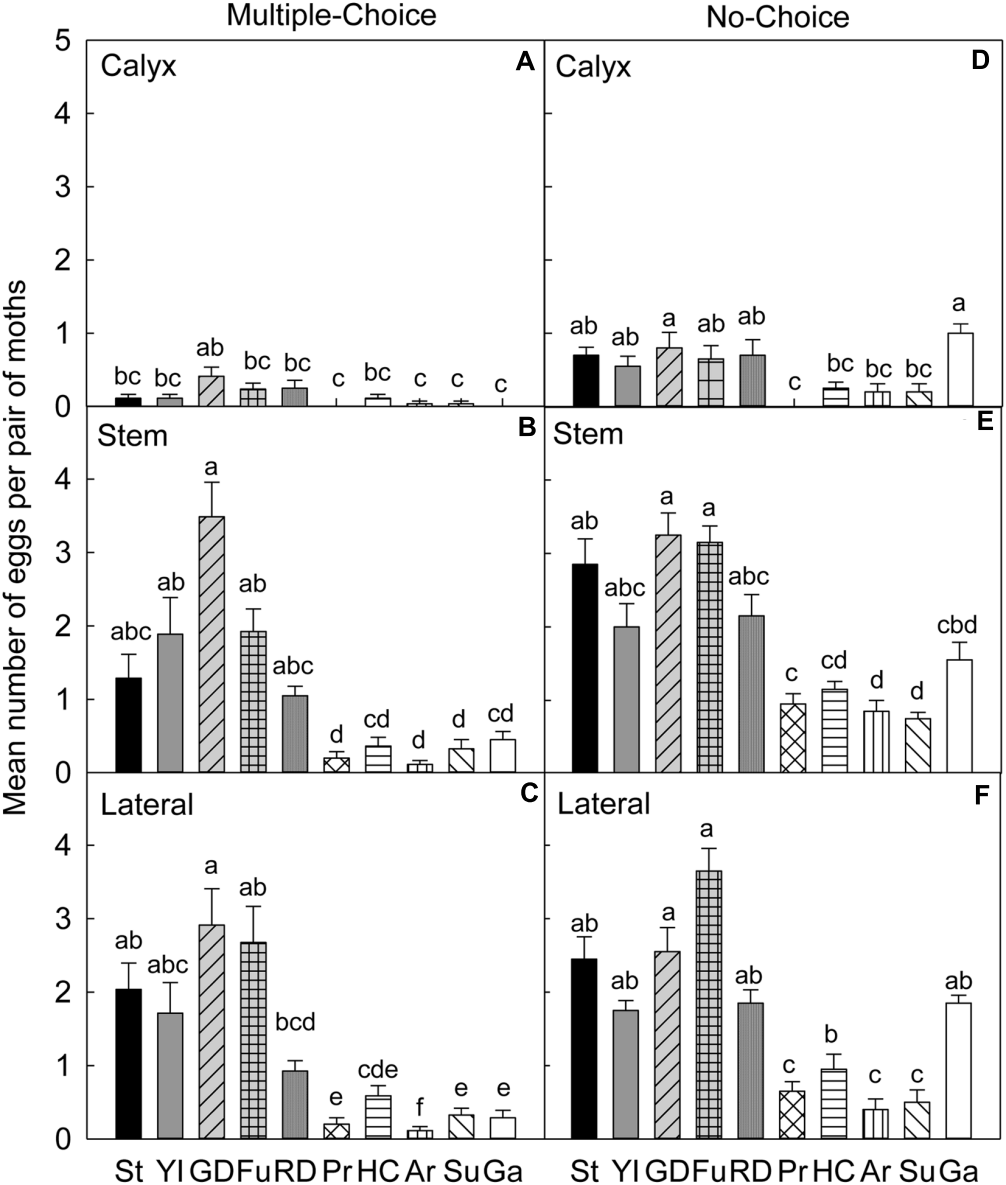
**Relative susceptibility of different apple cultivars for oviposition of codling moth during July 2007 (early season/Set 1).** Mean number of eggs per pair of moths per fruit on calyx, stem, and lateral sides of fruits of different cultivars are shown in multiple-choice tests **(A–C)** and no-choice tests **(D–F)**. St, Stayman; YI, York Imperial; GD, Golden Delicious; Fu, Fuji; RD, Delicious; Pr, Pristine; HC, Honeycrisp; Ar, Arlet; Su, Sunrise; Ga, Gala. *N* = 8 for all the multiple-choice tests, and *N* = 10 for all the no-choice tests. Each bar represents standard error of mean. Different letters over bars indicate significant difference (*P* < 0.05).

In the multiple-choice test of early season 2007, on the calyx site (**Table 4**), CM significantly preferred ‘Golden Delicious’ for oviposition over ‘Pristine’ (*P* = 0.006), ‘Arlet’ (*P* = 0.032), ‘Sunrise’ (*P* = 0.006), and ‘Gala’ (*P* = 0.032; **Figure 3A**). On the stem site of fruits, ‘Golden Delicious’ was again the significantly preferred cultivar for oviposition over other cultivars, *viz*., ‘Pristine’ (*P* < 0.001), ‘Honeycrisp’ (*P* < 0.001), ‘Arlet’ (*P* < 0.001), ‘Sunrise’ (*P* < 0.001), and ‘Gala’ (*P* < 0.001; **Figure 3B**). However, the preference for ‘Golden Delicious’ was similar to ‘Stayman’ (*P* = 0.092), ‘York Imperial’ (*P* = 0.457), ‘Fuji’ (*P* = 0.777), and ‘Delicious’ (*P* = 0.064; **Figure 3B**). On the lateral site, ‘Golden Delicious’ was significantly more preferred than all other cultivars (*P* < 0.05), except ‘Stayman’ (*P* = 0.996), ‘York Imperial’ (*P* = 0.777), and ‘Delicious’ (*P* = 0.109; **Figure 3C**). In contrast, ‘Arlet’ was the least preferred cultivar for oviposition on the lateral site of fruits (*P* < 0.05; **Figure 3C**).

In the no-choice test (July 2007), on the calyx site of fruits (**Table 4**), CM deposited higher numbers of eggs on ‘Golden Delicious’ and ‘Gala’ than on ‘Pristine,’ ‘Honeycrisp,’ ‘Arlet,’ and ‘Sunrise’ (*P* < 0.05; **Figure 3D**). On the stem (**Figure 3E**) and lateral (**Figure 3F**) sites of fruits, ‘Golden Delicious’ and ‘Fuji’ received the highest number of eggs over ‘Pristine,’ ‘Honeycrisp,’ ‘Arlet,’ and ‘Sunrise’ (*P* < 0.05).

### 2007 Late Season (August)

**FIGURE 4:**
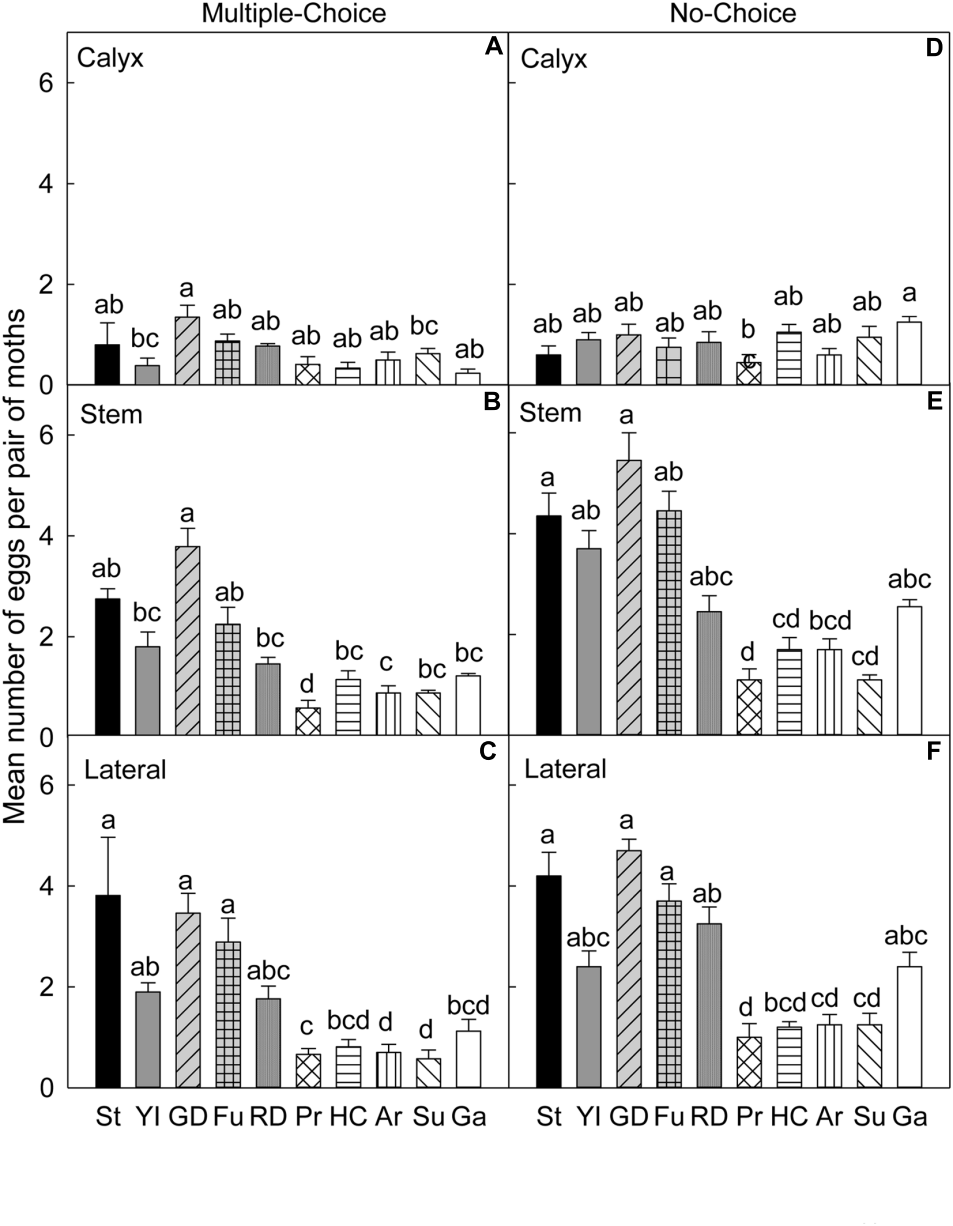
**Relative susceptibility of different apple cultivars for oviposition of codling moth during August 2007 (late season/Set 2).** Mean number of eggs per pair of moths per fruit on calyx, stem, and lateral sides of fruits of different cultivars are shown in multiple-choice tests **(A–C)** and no-choice tests **(D–F)**. St, Stayman; YI, York Imperial; GD, Golden Delicious; Fu, Fuji; RD, Delicious; Pr, Pristine; HC, Honeycrisp; Ar, Arlet; Su, Sunrise; Ga, Gala. *N* = 8 for all the multiple-choice tests, and *N* = 10 for all the no-choice tests. Each bar represents standard error of mean. Different letters over bars indicate significant difference (*P* < 0.05).

In the multiple-choice test conducted during the late season study of 2007, on the calyx site (**Table 4**), ‘Golden Delicious’ was more preferred for oviposition than ‘York Imperial’ (*P* = 0.036) and ‘Sunrise’ (*P* = 0.022; **Figure 4A**). On the stem site of fruits, the moths again preferred ‘Golden Delicious’ for oviposition over all other cultivars (*P* < 0.05), except ‘Stayman’ (*P* = 0.978) and ‘Fuji’ (*P* = 0.563; **Figure 4B**). In contrast, ‘Pristine’ was the least preferred cultivar (*P* < 0.05; **Figure 4B**). On the lateral site, ‘Stayman,’ ‘Golden Delicious,’ and ‘Fuji’ were the most preferred cultivars for oviposition (*P* < 0.05), except for ‘York Imperial’ and ‘Delicious’ (*P* > 0.05; **Figure 4C**).

In the no-choice test, on the calyx site (**Table 4**), CM deposited less eggs on ‘Pristine’ than ‘Gala’ (*P* = 0.047), however, such lower preference for ‘Pristine’ was not significantly different from all other cultivars (*P* > 0.05; **Figure 4D**). On the stem (**Figure 4E**) and lateral (**Figure 4F**) sites of fruits, CM showed less preference for ‘Pristine’ (*P* < 0.05) than all other cultivars, except for ‘Honeycrisp,’ ‘Arlet,’ and ‘Sunrise’ (*P* > 0.05).

### 2008 Early Season (July)

**FIGURE 5:**
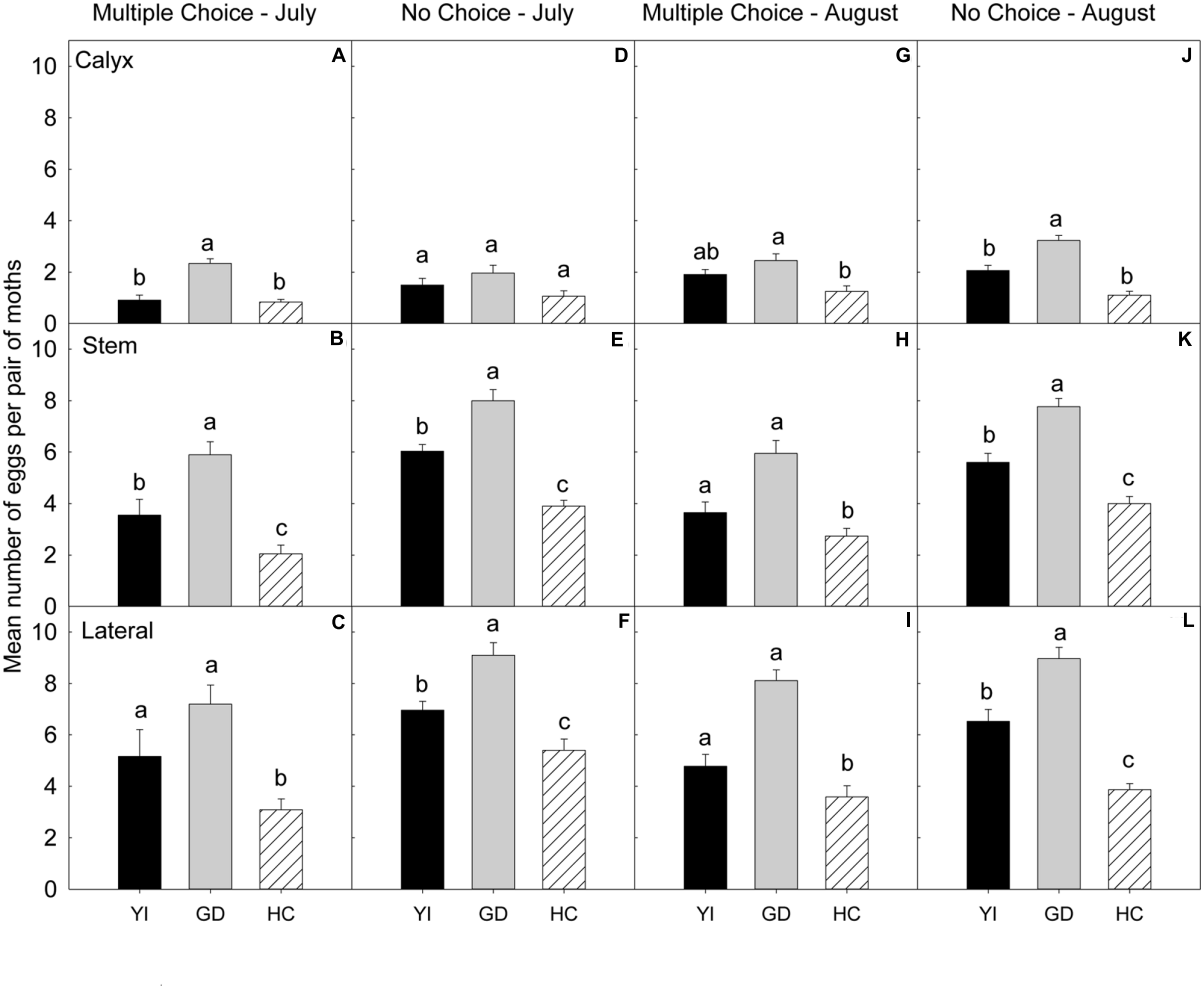
**Relative susceptibility of different apple cultivars for oviposition of codling moth during 2008.** Mean number of eggs per pair of moths per fruit on calyx, stem, and lateral sides of fruits of different cultivars are shown in multiple-choice tests- July 2008 **(A–C)** and August 2008 **(G–I)** and no-choice tests- July 2008 **(D–F)** and August 2008 **(J–L)**. YI, York Imperial, GD, Golden Delicious, HC, Honeycrisp. *N* = 8 for all the multiple-choice tests, and *N* = 15 for all the no-choice tests. Each bar represents standard error of mean. Different letters over bars indicate significant difference (*P* < 0.05).

In the multiple-choice test (**Table 4**), on the calyx (**Figure 5A**) and stem (**Figure 5B**) sites of fruits, ‘Golden Delicious’ was the most preferred cultivar for oviposition over the other two cultivars (*P* < 0.05). On the lateral site, ‘Honeycrisp’ was less preferred for oviposition than ‘Golden Delicious’ (*P* = 0.001; **Figure 5C**).

In the no-choice test (July 2008), on the calyx site (**Table 4**), the oviposition preference of CM did not differ significantly across all the cultivars (*P* > 0.05; **Figure 5D**). On stem (**Figure 5E**) and lateral sites of fruits (**Figure 5F**), CM deposited more eggs on ‘Golden Delicious’ over ‘Honeycrisp’ and ‘York Imperial’ (*P* < 0.05).

### 2008 Late Season (August)

In the multiple-choice test (**Table 4**), on the calyx site, ‘Golden Delicious’ was the most preferred cultivar over ‘Honeycrisp’ (*P* = 0.004), but it was not more preferred over ‘York Imperial’ (*P* = 0.475; **Figure 5G**). On stem (**Figure 5H**) and lateral (**Figure 5I**) sites of fruits, ‘Honeycrisp’ was the least preferred cultivar when compared to ‘Golden Delicious’ and ‘York Imperial’ (*P* < 0.05).

In the no-choice test (August 2008), on calyx (**Figure 5J**), stem (**Figure 5K**), and lateral (**Figure 5L**) sites of fruits, ‘Golden Delicious’ was the most preferred cultivar for oviposition over that of ‘Honeycrisp’ and ‘York Imperial’ (*P* < 0.05).

### Interaction Effects of Apple Cultivar, Choice (Type of Test), Season (Early or Late), Study Year and Oviposition Sites on CM Oviposition

All covariates (cultivar, season [early or late], and study year) had a significant influence on the oviposition of CM on different apple cultivars (*P* < 0.001; **Table 2**). All types of interactions presented in **Table 2** had a significant impact on the oviposition preference of CM (*P* < 0.05). Oviposition sites (i.e., calyx, stem, and lateral) on fruits had a highly significant influence on CM oviposition (*P* < 0.001; **Table 3**). All the interactions of oviposition sites with other covariates, except Site:Season (*P* = 0.458), Site:Cultivar:Season (*P* = 0.115) and Site:Cultivar:Year:Season (*P* = 0.615), displayed a significant interactive impact on the oviposition of CM (*P* < 0.05; **Table 3**).

### Susceptibility Index for Different Apple Cultivars for CM Oviposition

**FIGURE 6:**
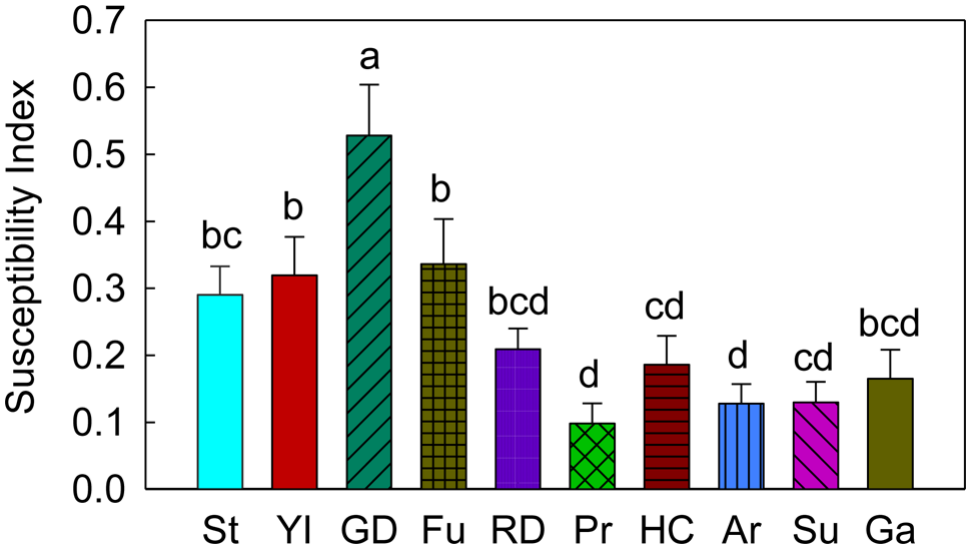
**Susceptibility index (SI) of different apple cultivars for oviposition of codling moth.** St, Stayman; YI, York Imperial; GD, Golden Delicious; Fu, Fuji; RD, Delicious; Pr, Pristine; HC, Honeycrisp; Ar, Arlet; Su, Sunrise; Ga, Gala. Each bar represents standard error of mean. *N* = 72 for all the cultivars, except GD, YI, and HC, where *N* = 95 and Pr and Ga, where *N* = 54. Error bar (standard error of mean) represents variability of oviposition in different cultivars across seasons (early and late), choices (no-choice and multiple-choice) and years (2006–2008). Different letters over bars indicate significant difference (*P* < 0.05).

In terms of the CM oviposition susceptibility index (on a scale of 0 – 1, where, ‘0’ = the least susceptible and ‘1’ = the most susceptible), ‘Golden Delicious’ had a significantly higher susceptibility index than ‘Stayman’ (*P* = 0.002), ‘York Imperial’ (*P* = 0.002), ‘Fuji’ (*P* = 0.011), ‘Delicious’ (*P* < 0.001), ‘Pristine’ (*P* < 0.001), ‘Honeycrisp’ (*P* < 0.001), ‘Arlet’(*P* < 0.001), ‘Sunrise’ (*P* < 0.001), and ‘Gala’ (*P* < 0.001; **Figure 6**). In contrast, ‘Pristine,’ ‘Honeycrisp,’ ‘Arlet,’ and ‘Sunrise’ were noticeably less susceptible to oviposition by CM (*P* < 0.05; **Figure 6**).

## Discussion

In the majority of bioassays conducted across different years, CM females preferred to oviposit on ‘Golden Delicious,’ ‘Fuji,’ ‘Delicious,’ ‘Stayman,’ and ‘York Imperial’ over other cultivars, *viz*., ‘Pristine,’ ‘Honeycrisp,’ ‘Arlet,’ ‘Sunrise,’ and ‘Gala.’ Different volatile fruit-coat constituents likely affect the ovipositional preferences by CM for apple fruits. For instance, the production of the sesquiterpene α-farnesene, an ovipositional stimulant for female CM, and an important constituent in the outer skin of apple fruits, varies greatly across different cultivars and changes as fruit mature (Wearing and Hutchins, 1973; Sutherland et al., 1977). Such variation could be an important factor in helping explain the differential cultivar ovipositional preferences of CM found in this study. However, such hypothesis needs further evaluation.

Results from the no-choice and multiple-choice tests across different years in this study showed that CM females deposited significantly more eggs on ‘Golden Delicious’ over other cultivars, *viz*., ‘Pristine,’ ‘Honeycrisp,’ ‘Arlet,’ and ‘Sunrise.’ Similar trends in the ovipositional preferences of a closely related tortricid pest, oriental fruit moth [*Grapholita molesta* (Busck)] for these different apple cultivars are also reported (Joshi et al., 2007; Myers et al., 2007). In the one study by Joshi et al. (2007), the oriental fruit moth preferred ‘Golden Delicious’ for oviposition compared to the cultivars ‘Pristine,’ ‘Arlet,’ and ‘Sunrise.’ Based on the ovipositional preferences exhibited by CM, these preferred cultivars are highly likely more susceptible to CM infestations, especially if these laboratory results reflect field behaviors. The choice of a preferred suitable substrate or host for oviposition plays a key role in the survival and completion of different life stages of lepidopteran insects (Chew and Robbins, 1984; Renwick, 1989). Similarly, in the case of CM, judicious selection of an appropriate host for depositing eggs might play a key role in determining the initial fate of a neonate larva that feeds internally in fruits of the selected host(s). Upon hatching, the larva enters the fruit, and remains inside the fruit till the pre-pupal stage. The larva developing inside the fruit is usually incapable of moving from one fruit to other, so the oviposition preferences of female CM most likely determine larval survival by selecting the most suitable host/cultivar. In oviposition preference studies of a closely related fruit pest species (i.e., oriental fruit moth), Myers et al. (2006a) found higher percentages of larval entry in fruits of preferred (in terms of oviposition) cultivars like ‘Golden Delicious’ and ‘Delicious’ during their early and late season experiments. Therefore, it is likely that the oviposition preference of CM for these different apple cultivars might be related to larval survival. The percent larval survival on the most preferred cultivar (i.e., ‘Golden Delicious’) was higher than one of the less preferred cultivars (i.e., ‘Arlet’) when neonate larvae were individually exposed to these different cultivars (NKJ et al., unpublished data). Such preferences for ‘Golden Delicious’ were also revealed in the present oviposition bioassays, as ‘Golden Delicious’ was the preferred cultivar over ‘Arlet,’ ‘Sunrise,’ and ‘Pristine’ cultivars. In a related study on relative susceptibility of different apple cultivars to various arthropod pests that was conducted in an orchard, Hogmire and Miller (2005) reported ‘Golden Delicious’ as a highly susceptible cultivar to CM infestations versus other cultivars such as ‘Pristine,’ ‘Honeycrisp,’ ‘Arlet,’ and ‘Sunrise.’

Early maturing varieties have been considered less susceptible to CM infestations (Isely, 1943). In the present study, for the majority of oviposition bioassays, CM least preferred to oviposit on early maturing cultivars, *viz*., ‘Pristine,’ ‘Honeycrisp,’ ‘Arlet,’ ‘Sunrise,’ and ‘Gala’ as compared to later maturing cultivars such as ‘Stayman,’ ‘York Imperial,’ ‘Golden Delicious,’ ‘Fuji,’ and ‘Delicious.’ Such preference could be related to the presence or emission of fruit volatiles from these cultivars, since fruit volatiles are known to play a crucial role in guiding female moths to oviposit on or near fruits (Wildbolz, 1958; Lombarkia and Derridj, 2002; Reed and Landolt, 2002). The oviposition preferences of CM across different cultivars may also vary in relation to the time during the season that an apple matures and to its fruit maturity at any specified time during the season, because the release of volatiles from fruits increases from early to late season (Sutherland et al., 1977; Mattheis et al., 1991). In general, more eggs per pair of CM adults per fruit were observed in bioassays conducted with fruits collected later in the season (August) than those collected earlier in the season (July). During the early stages of fruit development, fruits are reported to release only a few ester type compounds as compared to ripening and mature fruits (late season), which are reported to release many ester type compounds plus a few terpenoids (Bengtsson et al., 2001). Such changes in volatile emissions may be the reason for the variations in the ovipositional preferences of CM for fruits collected in July (early season) and August (late season). During the early stages of fruit development, CM females are reported to deposit more eggs on neighboring leaves (i.e., shoot and spur) around fruits and fruit clusters (Wildbolz, 1958; Blomfield et al., 1997) than directly on fruits (NKJ et al. unpublished data), while during the fruit maturation and ripening period, more eggs are deposited directly on fruits compared to the early stages of fruit development (Summerland and Steiner, 1943). This type of oviposition pattern/preference may be helpful in increasing the likelihood of larval survival upon hatching. Sutherland et al. (1977) found that the production of α-farnesene (which is known to influence the oviposition behavior of CM) increases as fruit maturity increases. Consequently, CM females deposit more eggs on fruits as fruit maturity increases during the season. The variability among different apple cultivars in the production of the oviposition stimulant α-farnesene could be a major factor affecting the ovipositional preference of CM for different apple cultivars during the early and latter part of the growing season. Other strong possibilities causing such early and late season variation in the oviposition preferences of CM could be the differential developmental stages (maturity level) and other characteristics (such as chemical composition of fruit-coat, fruit color, etc.) of fruits of these different cultivars during the two different time periods of a season.

Apart from the chemical constituents of the fruit coat, physical characteristics of the apple fruit surface may vary from one cultivar to other, as well as within the calyx, stem and lateral sides of fruit of different cultivars (Belding et al., 1998; Verardo et al., 2003). Such microtopographic properties can be categorized on the basis of roughness and smoothness of host surface, and play a crucial role in the attachment ability of CM (Al Bitar et al., 2010), and may influence its oviposition behavior, particularly the oviposition site selection (Al Bitar et al., 2014). Friction forces, which affect the attachment ability of CM eggs to these different types of surfaces, had been reported to be higher on oviposition substrates with smooth surfaces (Al Bitar et al., 2009, 2010), and could be main factors behind the CM oviposition preferences for the smooth substrates (e.g., fruits) over rough surfaces (e.g., leaves with trichomes). Variation in the CM oviposition on calyx, stem and lateral sides of fruits of apple cultivars in this study could be due to differences in fruit surface properties such as amorphous wax layer (comprised of microcracks and epicuticular wax crystals) favoring CM egg adhesion to oviposition substrates. Composition and abundance of microcracks (Al Bitar et al., 2014) and epicticular wax (Belding et al., 1998) on fruit surfaces vary across different apple cultivars. CM egg adhesion to different oviposition substrates of the fruit of different cultivars (for instance, ‘Golden Delicious,’ ‘Elstar,’ ‘Jonica,’ ‘Boskoop,’ ‘Topaz’) had been reported to vary within upper (stem), middle (lateral), and lower (calyx) sections of fruits (Al Bitar et al., 2014). Regardless of test type, year and season, in general, we recorded higher number of eggs on stem and lateral sites compared to calyx end of fruit. It could be due to higher abundance of microcracks as well as stronger bonding between CM eggs and fruit surfaces on stem and lateral fruit surfaces than calyx end (Al Bitar et al., 2014).

Cultivar, season (early or late), study year and oviposition sites (i.e., calyx, stem, and lateral) on fruits had a significant influence on the oviposition of CM on different apple cultivars. Covariate interactions (except, Site:Season, Site:Cultivar:Season, and Site:Cultivar:Year:Season) were also significant. Oviposition-sites on fruits may vary from one cultivar to another. In the multiple-choice and no-choice tests, CM deposited more eggs on lateral and stem sites than on the calyx site of fruits. Such patterns of egg deposition could be due to the physical characteristics of the apple fruit surface as discussed earlier or due to the ‘vertical’ placement of fruits, as in all these tests, fruits were vertically placed in oviposition chambers. In contrast, oriental fruit moth adult females preferred to oviposit on the calyx and stem sites of apple fruit, and their oviposition site preferences are also reported to vary between different apple cultivars (Myers et al., 2006b).

Susceptibility to various pest infestations may vary among cultivated varieties as well as wild varieties (e.g., crab apples). In the past, susceptibility of apple cultivars/germplasms to different arthropod pests has been studied using several methods, such as their impact on pest developmental rate and pest survival rate (Mackenzie and Cummins, 1982; Myers et al., 2006b), damage (in terms of fruit injury) caused by pests (Dean and Chapman, 1973; Goonewardene et al., 1979; Straub, 2003; Hogmire and Miller, 2005) and the occurrence of pests (Goonewardene et al., 1976; Straub, 2003; Hogmire and Miller, 2005; Myers et al., 2007). However, using a standardized oviposition-based susceptibility index of apple cultivars as developed in this study reveals important information about the relative susceptibility of cultivars when evaluated under different seasons and times during the season. The newly developed CM oviposition susceptibility index for apple cultivars showed that susceptibility is linked to the oviposition preferences of CM, as female moths least preferred ‘Pristine,’ ‘Sunrise,’ ‘Arlet,’ and ‘Honeycrisp’ (less susceptible cultivars) for oviposition than ‘Golden Delicious’ (highly susceptible cultivar). Similarly, Hogmire and Miller (2005) reported that ‘Golden Delicious’ was significantly more susceptible than ‘Pristine,’ ‘Honeycrisp,’ ‘Arlet,’ and ‘Sunrise’ to injury by CM in the field environment. Straub (2003) studied the relative susceptibility of some new apple cultivars in New York to different orchard pests, and found that cultivars such as ‘Sunrise,’ ‘Pristine,’ ‘McIntosh’ (Pioneer), and ‘Honeycrisp’ were comparatively resistant to CM larval damage compared to ‘Golden Delicious’. The CM oviposition susceptibility index could be useful to researchers/research extension workers and fruit growers in IPM decision-making in apple orchards.

To summarize, CM preferred to oviposit on later maturing cultivars ‘Golden Delicious,’ ‘Stayman,’ ‘York Imperial,’ ‘Fuji,’ and ‘Delicious’ (preferred cultivars) than early maturing cultivars, *viz*., ‘Pristine,’ ‘Honeycrisp,’ ‘Arlet,’ ‘Sunrise,’ and ‘Gala’ (less preferred cultivars) in the majority of the multiple-choice tests. In the no-choice tests, CM deposited more eggs on these preferred cultivars than the less preferred cultivars. Regardless of choice test type and season, CM deposited significantly more eggs on ‘Golden Delicious’ over other cultivars, *viz*., ‘Pristine,’ ‘Honeycrisp,’ ‘Arlet,’ and ‘Sunrise.’ In both types of tests, more eggs were laid on lateral and stem sites than the calyx site of fruits across different cultivars. In terms of a CM oviposition susceptibility index, ‘Golden Delicious’ was the most susceptible cultivar to oviposition, while ‘Pristine,’ ‘Honeycrisp,’ ‘Arlet,’ and ‘Sunrise’ were least susceptible. From an integrated pest management perspective, the newly developed susceptibility index can assist fruit growers and consultants select the most appropriate cultivar(s) for monitoring and detecting the initial signs of fruit injury from this pest. For instance, ‘Golden Delicious’ is the most preferred cultivar for oviposition, therefore it should be the cultivar of choice for monitoring CM injury in mixed-cultivar planted orchards. If it is not present in a block/orchard, then the next preferred cultivar for oviposition should be selected for examining CM injury or oviposition. In addition, results of these studies would be helpful in breeding programs, particularly in developing CM resistant apple varieties. As previously discussed, oviposition by CM is likely stimulated by fruit volatiles, and variations in the production and release of these volatiles from different apple cultivars may result in different oviposition preferences. Further investigations are needed to understand the biochemical as well as physical aspects of fruits and other factors involved in determining apple cultivar susceptibility for CM oviposition.

## Conflict of Interest Statement

The authors declare that the research was conducted in the absence of any commercial or financial relationships that could be construed as a potential conflict of interest.
